# Statins and Selective Inhibition of Rho Kinase Protect Small Conductance Calcium-Activated Potassium Channel Function (K_Ca_2.3) in Cerebral Arteries

**DOI:** 10.1371/journal.pone.0046735

**Published:** 2012-10-08

**Authors:** Alister J. McNeish, Francesc Jimenez-Altayo, Graeme S. Cottrell, Christopher J. Garland

**Affiliations:** 1 Reading School of Pharmacy, University of Reading, Reading, Berkshire, United Kingdom; 2 Department of Pharmacology, University of Oxford, Oxford, Oxfordshire, United Kingdom; 3 Department of Pharmacy and Pharmacology, University of Bath, Bath, Bath and North East Somerset, United Kingdom; The Chinese University of Hong Kong, Hong Kong

## Abstract

**Background:**

In rat middle cerebral and mesenteric arteries the K_Ca_2.3 component of endothelium-dependent hyperpolarization (EDH) is lost following stimulation of thromboxane (TP) receptors, an effect that may contribute to the endothelial dysfunction associated with cardiovascular disease. In cerebral arteries, K_Ca_2.3 loss is associated with NO synthase inhibition, but is restored if TP receptors are blocked. The Rho/Rho kinase pathway is central for TP signalling and statins indirectly inhibit this pathway. The possibility that Rho kinase inhibition and statins sustain K_Ca_2.3 hyperpolarization was investigated in rat middle cerebral arteries (MCA).

**Methods:**

MCAs were mounted in a wire myograph. The PAR2 agonist, SLIGRL was used to stimulate EDH responses, assessed by simultaneous measurement of smooth muscle membrane potential and tension. TP expression was assessed with rt-PCR and immunofluorescence.

**Results:**

Immunofluorescence detected TP in the endothelial cell layer of MCA. Vasoconstriction to the TP agonist, U46619 was reduced by Rho kinase inhibition. TP receptor stimulation lead to loss of K_Ca_2.3 mediated hyperpolarization, an effect that was reversed by Rho kinase inhibitors or simvastatin. K_Ca_2.3 activity was lost in L-NAME-treated arteries, but was restored by Rho kinase inhibition or statin treatment. The restorative effect of simvastatin was blocked after incubation with geranylgeranyl-pyrophosphate to circumvent loss of isoprenylation.

**Conclusions:**

Rho/Rho kinase signalling following TP stimulation and L-NAME regulates endothelial cell K_Ca_2.3 function. The ability of statins to prevent isoprenylation and perhaps inhibit of Rho restores/protects the input of K_Ca_2.3 to EDH in the MCA, and represents a beneficial pleiotropic effect of statin treatment.

## Introduction

In rat middle cerebral arteries (MCA) endothelium-dependent hyperpolarization (EDH) responses (commonly called endothelium derived hyperpolarizing factor, EDHF, response) are observed in the presence of NO synthase (NOS) inhibitors, and can be abolished by inhibition of endothelial cell K_Ca_3.1 (intermediate conductance, IK_Ca_) channels, irrespective of the agonist used to stimulate EDH [1], [2]. In most other arterial beds, inhibition of both endothelial cell K_Ca_3.1 and K_Ca_2.3 (small conductance, SK_Ca_) is necessary for block of EDH [3]. However, the MCA does expresses endothelial cell K_Ca_2.3 [4], [5] which contribute to EDH in vessels still able to synthesise NO [5]. Following inhibition of NO synthase, input from K_Ca_2.3 to EDH responses is restored in the middle cerebral artery by exposure to antagonists of thromboxane receptors (TP) [6]. As TP stimulation suppresses the K_Ca_2.3 input to EDH in rat middle cerebral and mesenteric arteries [6], [7], endogenous stimulation may represent a significant influence on K_Ca_2.3 function in the vasculature. The mechanism that ‘protects’ K_Ca_2.3 function during NO signalling or TP inhibition remains unclear.

NO could potentially protect K_Ca_2.3 channel function by direct interaction/stimulation of the channel [8]. Alternatively, NO might inhibit the synthesis of metabolites that affect K_Ca_ channels by binding to the heme groups of enzymes. For example, the cytochrome P450 metabolite 20-HETE inhibits EDH responses in coronary arteries [9]. Neither of these pathways is likely to explain the ‘protective’ effect of NO in cerebral arteries, as hyperpolarization evoked by exogenous NO is inhibited by the KCa1.1 blocker iberiotoxin and therefore does not involve K_Ca_2.3 [10] and inhibition of 20-HETE synthesis did not influence K_Ca_2.3 function [6]. However, as K_Ca_2.3 function is restored by antagonizing TP [6], NO may protect K_Ca_2.3 function by PKG dependent inhibition of these receptors [11] or by inhibiting the generation of metabolites that could stimulate this receptor by binding to heme groups [12].

A major signalling pathway associated with TP is activation of Rho kinase [13]. TP are expressed primarily on the smooth muscle cell layer but they can also be expressed in endothelial cells [14]. It is likely that TP signalling in endothelial cells also involves Rho kinase therefore they may regulate the K_Ca_2.3 channels expressed selectively in these cells. The role of Rho kinase signalling on K_Ca_2.3 channel function can be directly assessed using inhibitors of this kinase but the statin class of drugs are also reported to have effects on Rho mediated signalling. They improve endothelium-dependent relaxation via a mechanism that involves inhibition of Rho signalling [15], independently of their ability to lower cholesterol.

The aims of the current study were 1) to investigate if disrupting the Rho kinase pathway could protect K_Ca_2.3 functionality following TP stimulation; 2) to establish if inhibition of Rho kinase signalling might restore the K_Ca_2.3 component of the EDH response suppressed by the presence of NOS inhibitors, and 3) to assess if statins had a similar effects to inhibitors of Rho kinase.

## Materials and Methods

### Animals and Ethics Statement

Male Wistar rats (200–300 g) were humanely killed by cervical dislocation following institutional guidelines for animal welfare and schedule 1 of the Animals (scientific procedures) Act 1986. The brain was removed and immediately placed in ice-cold Krebs solution. Segments of the MCA (∼2 mm long) were dissected and stored in ice-cold Krebs for use within 30 min, with similar size vessels used in all experimental groups.

### Simultaneous Measurement of Membrane Potential and Tension

Segments of MCA (internal diameter ∼150 µm) were mounted in a Mulvany-Halpern myograph (model 400A, Danish Myotechnology) in Krebs solution containing (mM): NaCl, 118.0; NaCO_3_, 24; KCl, 3.6; KH_2_PO_4_, 1.2; MgSO_4_⋅7H_2_O, 1.2; glucose, 11.0; CaCl_2_, 2.5; gassed with 95% O_2_, 5% CO_2_ and maintained at 37°C. After equilibration for 20 min, vessels were tensioned to 1–1.5 mN (approximates wall tension at 60 mmHg). Smooth muscle tension was recorded with an isometric force transducer and Powerlab software (ADI, Australia). Vessel viability was assessed by adding exogenous K^+^ (15–55 mM, total K^+^ concentration); only vessels developing tension of ≥3 mN were used. Endothelial cell viability was assessed by the ability of the agonist of protease-activated receptor 2 (PAR2) SLIGRL (serine, leucine, isoleucine, glycine, arginine, leucine-NH_2_; 20 µM) to relax spontaneous tone and to hyperpolarize the smooth muscle cell membrane by >15 mV. A concentration of 20 µM SLIGRL was used in all further experiments; our previous study characterized the potency of this agent to evoke endothelium dependent relaxation and this concentration evokes circa 75–80% of the maximal relaxation response [1]. Use of this single concentration also minimises potential desensitization of the PAR2 receptor.

In some experiments endothelium-dependent responses to SLIGRL were obtained in vessels that were able to synthesize NO but were pre-constricted to approximately 80% of maximal tone (obtained with 55 mM K^+^) with varying concentrations of the TP receptor agonist U46619 (circa 50–100 nM). Under these conditions EDH was assessed in the presence of the K_Ca_ channel blockers used at concentrations that should cause full channel inhibition with no off target effects at other K_Ca_ channels with respect to their published K_i_ values, apamin (K_Ca_2.3, 100 nM) [16], TRAM-34 (K_Ca_3.1, 1 µM) [17] and iberiotoxin (K_Ca_1.1, 100 nM) [18] Previous studies have shown that the order in which these drugs are added has no effect on the experimental outcome [1], [5]. For clarity of presentation they were always added in the order: 1) TRAM-34; 2) The combination of TRAM-34 and apamin; 3) the combination of TRAM-34, apamin and iberiotoxin. The effect of K_Ca_ blockers on EDH responses was also assessed after addition of inhibitors of Rho kinase (Y27632; 1 and 10 µM) [19], and HMG-CoA reductase (simvastatin; 0.1 and 1 µM). We did not routinely measure relaxation in vessels able to synthesise NO, under these conditions maximal vasodilatation due to NO release persists after hyperpolarization is blocked [5]. In all other experiments, isolated EDH responses were obtained in the presence of the NO synthase inhibitor L-NAME (100 µM). We have previously reported that under these conditions only K_Ca_3.1 contribute to EDH [1] thus the other K_Ca_ channel blockers were added in the sequence detailed above only if the previous K_Ca_ blocker(s) failed to prevent hyperpolarization. K_Ca_ channel blockers were assessed against EDH responses to SLIGRL (20 µM) in the presence of: 1. the Rho kinase inhibitors Y27632 (10 µM) [19] or SR5037 (1 µM) [20] as no kinase inhibitor is 100% selective we chose two structurally distinct inhibitors at concentrations approximately 100 times their respective Ki/IC_50_ for Rho kinase to ensure full inhibition with minimal effects on other, related kinases [19], [20]; 2. the HMG-CoA inhibitors simvastatin (0.1 and 1 µM) or lovastatin (100 nM).These concentrations of statins are similar to the concentrations found in human plasma (circa 25–90 nM) [21] and are in line with their K_i_ values of low to high nanomolar, reported in various rat cell types [22] In some experiments, in the presence of L-NAME and simvastatin (100 nM) the vessels were also incubated with the isoprenoid geranylgeranyl-pyrophosphate (GGPP, 1 µM). The concentration of GGPP used corresponds to those reported to reverse statin mediated inhibition of RhoA in vascular endothelial cells [15], [23] Papaverine (150 µM) was added at the end of each experiment to assess overall tone (previous experiments show this was sufficient to give a maximal relaxation indistinguishable from that obtained in the presence of zero calcium [24]. All drugs were allowed to equilibrate for 20 min before vasodilator responses were stimulated. The statins and GGPP were incubated for >60 min; longer than prenylation-dependent translocation of Rho measured in cerebral artery smooth muscle cells [25]. In most experiments smooth muscle membrane potential (E_m_) and tension were measured simultaneously (except where stated) at a sample rate of 100 Hz as previously described, using glass microelectrodes (filled with 2 M KCl; tip resistance, 80–120 MΩ) to measure E_m_ [26].

### Reverse Transcription-PCR

RNA from intact rat MCAs was isolated using Trizol (Life Technologies Ltd., Paisley, UK) and was reverse-transcribed using standard protocols with random hexamers and TaqMan reverse transcription reagents (Life Technologies Ltd.). Subsequent PCR reactions used primers specific for rat TP receptor (forward 5′- CTCCTGGTGCTTCTTGACTC-3′; reverse 5′-GAGCTGGGAAGTGAACCTTG-3′) and K_Ca_2.3 (forward 5′-ACTTCAACACCCGATTCGTC-3′; reverse 5′- TTGACACCCCTCAGTTGGTG-3′). Control reactions omitted reverse transcriptase. PCR products were separated by electrophoresis, stained with ethidium bromide, and sequenced to confirm identity.

### Immunofluorescence

Sources of antibodies were as follows: mouse anti-rat Platelet/endothelial cell adhesion molecule-1 (PECAM-1/CD31, TLD-3A12, Millipore, Watford, UK); rabbit anti-mouse thromboxane A2 receptor (101882; Cayman, Tallinn, Estonia) and donkey anti-mouse Cy5 and anti-rabbit Rhodamine Red-X (Stratech Scientific Limited, Newmarket, UK).

Arteries were cut longitudinally and pinned flat on sylgard plates and fixed with 100 mM PBS, pH 7.4 containing 4% paraformaldehyde (30 min, 4°C). Artery whole mounts were washed in PBS containing 10% normal horse serum and 0.3% Triton-X-100 (1 h, room temperature) and proteins localized using primary antibodies: Thromboxane A2 receptor (1∶100) and PECAM-1 (CD31, 1∶200) (24 h, 4°C). Whole mounts were washed (1 h, room temperature), incubated with secondary antibodies coupled to Rhodamine Red-X, or Cy5 (1∶500, 2 h, room temperature) and mounted in ProLong® Gold (Invitrogen, Paisley, UK) which contains DAPI.

Whole mounts were observed with a Zeiss laser-scanning confocal microscope (LSM Meta 510) using an EC Plan-Neofluar 40×/1.3 Oil DIC objective. Images were collected at a zoom of 1.5 and an iris of <3 µm, and at least five optical sections were taken at intervals of 0.5 µm. Single sections are shown. Images were processed using ImageJ and Adobe Photoshop software. The specificity of the immunostaining was evaluated by omission of the primary antibody and processed as above. Under these conditions, no staining was observed in any experimental situation.

### Data Analysis and Statistical Procedure

Results are expressed as the mean±s.e.mean of *n* animals. Tension values are given in mN (always per 2 mm segment) and E_m_ as mV. Vasodilatation is expressed as percentage reduction of the total vascular tone (spontaneous tone plus vasoconstrictor response), quantified by relaxation with papaverine (150 µM). Graphs were drawn and comparisons made using one-way ANOVA with Tukeys’ post-test (Prism, Graphpad). P≤0.05 was considered significant. pEC_50_ values were calculated using non-linear regression fitted to the Hill equation with variable slope (Prism, Graphpad).

### Drugs, Chemicals, Reagents and Other Materials

Exogenous K^+^ was added as an isotonic physiological salt solution in which all the NaCl was replaced with an equivalent amount of KCl. Concentrations of K^+^ used are expressed as final bath concentration. GGPP, L-NAME (N^G^-nitro-L-arginine methyl ester), papaverine HCl, TRAM-34 (1-[(2-Chlorophenyl)diphenylmethyl]-1H-pyrazole), U46619 (9,11-Dideoxy-11α,9α-epoxymethanoprostaglandin F2α) and Y27632 ((R)-(+)-trans-4-(1-aminoethyl)-N-(4-pyridyl) cyclohexane carboxamide dihydrochloride) were all obtained from Sigma (Poole, U.K.). Apamin and iberiotoxin, from Latoxan (Valence, France). SLIGRL-NH2 from Auspep (Parkville, Australia). Simvastatin and Lovastatin were from Cayman-Europe (Tallinn, Estonia). SR5037 ((R)-6-Methoxy-N-(2-(1-methylpiperidin-4-yloxy)-4-(1H-pyrazol-4-yl)phenyl)-1,2,3,4-tetrahydroisoquinoline-3-carboxamide) was a generous gift from Professor Phil LoGrasso (Scripps Research Institute, Florida, USA). All stock solutions (100 mM) were prepared in dimethylsulfoxide except L-NAME, apamin, iberiotoxin, papaverine and SLIGRL which were dissolved in 0.9% NaCl. Controls included appropriate vehicle. All blocking drugs were used at appropriate concentrations to ensure full inhibition of their target (normally 10–100 times the Ki/IC_50_ value) and minimal off target effects and are the recommended selective inhibitors as classified by IUPHAR [27].

## Results

### Thromboxane Receptors are Expressed in Endothelial Cells of Middle Cerebral Arteries

**Figure 1:**
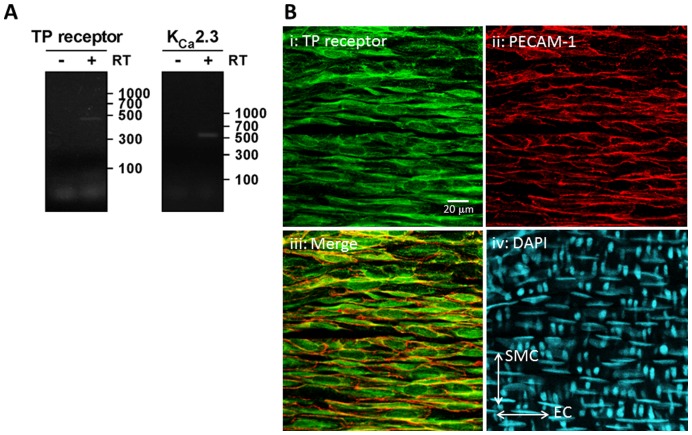
Expression of the TP receptor in the endothelium of rat middle cerebral arteries. A) RT-PCR amplification of mRNA transcripts for TP receptor (439 bp) and K_Ca_2.3 channels (514 bp) from rat MCA. bp = base pairs B) Localization of TP receptor and PECAM-1-immunoreactivity in whole mount preparations of rat MCA. TP receptor-immunoreactivity was present in the endothelial cell layer (PECAM-1-positive). Orientation of cell nuclei was determined using DAPI. The merged image demonstrates coexpression of TP receptors and PECAM-1, indicating TP receptor expression in the endothelial cells of rat MCAs. Scale bar, 20 µm.

While TP receptors are known to be expressed in human endothelial cells derived from umbilical or saphenous veins [14], expression has never been demonstrated in the endothelium of the rat MCA. We examined expression of TP receptors and K_Ca_2.3 in the MCA by RT-PCR (Figure 1A). We amplified mRNA transcripts of expected sizes and confirmed identity by sequencing. Next, we examined expression of TP receptors in whole mount preparations of rat MCA using immunofluorescence and confocal microscopy. We detected TP receptor-immunoreactivity in the endothelial cell layer (PECAM-1-positive) of rat MCA (Figure 1B–E). Nuclei orientation was confirmed using DAPI, with endothelial cell nuclei orientated in the horizontal plane, smooth muscle cell nuclei in the vertical. TP receptor-immunoreactivity was also detected in the smooth muscle cell layer (PECAM-1-negative) (data not shown). Together, these data suggest that TP receptors are appropriately localized on endothelial cells to regulate K_Ca_2.3 channel function.

### Inhibiting Rho Kinase Prevents Loss of K_Ca_2.3 Input to EDH during TP Receptor Stimulation

**Figure 2:**
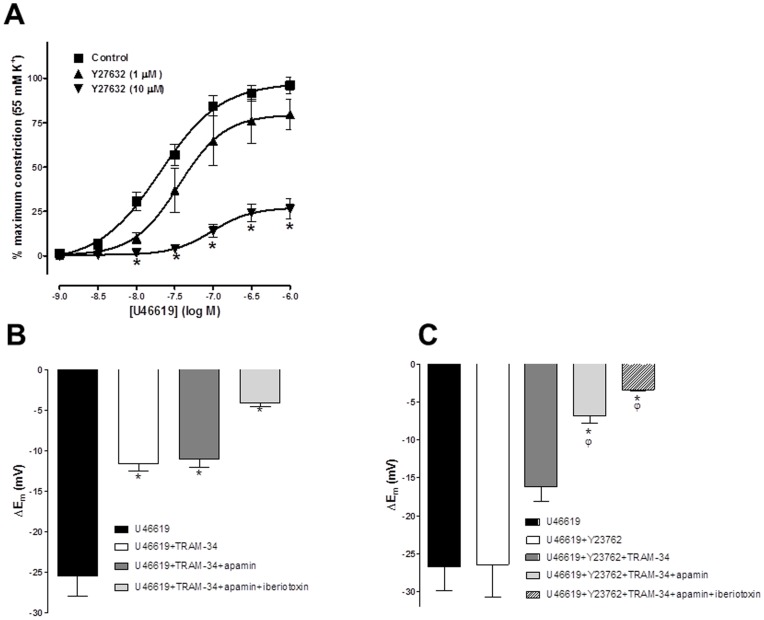
Effect of inhibition of Rho Kinase on TP induced constriction and EDH responses obtained in the presence of TP stimulation in the rat middle cerebral artery. (A) Concentration response curve showing the vasoconstrictor response produced by the thromboxane A_2_ mimetic U46619 (1 nM-1 µM; n = 5) in rat middle cerebral arteries in the presence and absence of the selective Rho kinase inhibitor Y27632 (1 and 10 µM; n = 5). Inhibition of Rho kinase significantly and concentration dependently reduced the maximum constriction produced by U46619 and significantly shifted the concentration response curve to the right. (B) Histogram showing SLIGRL (20 µM) induced EDH evoked in the presence of U46619 (50–100 nM) in vessels able to synthesise NO. Also shown are EDH in the presence of K_Ca_3.1 blockade (1 µM, TRAM-34), blockade of both K_Ca_2.3 and 3.1 (100 nM apamin+TRAM-34) and blockade of K_Ca_1.1, 2.3 and 3.1 (100 nM Iberiotoxin+apamin+TRAM-34). Block of K_Ca_3.1 alone was sufficient to significantly reduce EDH; subsequent block of K_Ca_2.3 had no further effect indicating this channel was not functional. Residual EDH was inhibited by further blockade of K_Ca_1.1. (C) Histogram showing SLIGRL induced EDH in the presence of U46619 and the Rho Kinase inhibitor Y27632 in vessels able to synthesise NO. EDH was only significantly reduced following combined blockade of both K_Ca_3.1 and 2.3, indicating that the K_Ca_2.3 channel was now functional. *P<0.05 indicates a significant difference from control (U46619 alone) using one-way ANOVA with Tukey’s post-test, n = 5–6) ^φ^P<0.05 indicates a significant difference from Y27632 as determined by one-way ANOVA with Tukey’s post-test, n = 5–6.

In the absence of a NO synthase inhibitor, the thromboxane mimetic U46619 evoked concentration dependent constriction (pEC_50_ = 7.69±0.08, n = 5; Figure 2A). The selective Rho kinase inhibitor Y27632 concentration-dependently inhibited this response (pEC_50_ = 7.45±0.15 and 7.02±0.14, in presence of 1 and 10 µM Y27632, respectively, n = 5, P<0.05, Figure 2A). Y27632 (10 µM) also significantly reduced the maximum constriction produced by U46619 (control, 96.1±4.6%; 10 µM Y27632, 26.4±5.7%, n = 5, P<0.05; Figure 2A).

When MCAs are able to synthesise NO, EDH is mediated by both K_Ca_2.3 and K_Ca_3.1 with a small NO mediated K_Ca_1.1 component [6], [10]. Under similar conditions in the present study, excepting that 100 nM U46619 was present to stimulate TP receptors, SLIGRL-evoked EDH (25.4±2.5 mV, n = 5) was partially blocked by the K_Ca_3.1 inhibitor TRAM-34 (1 µM; hyperpolarization of 11.6±0.9 mV, n = 5, P<0.05) and the subsequent application of the K_Ca_2.3 blocker apamin (100 nM) had no further effect (11.0±1.1 mV, n = 5). However, the residual EDH was suppressed by the K_Ca_1.1 (BK_Ca_) blocker, iberiotoxin (100 nM; 4.0±0.5 mV, n = 5; Figure 2B). In the presence of U46619 if arteries were exposed to the Rho kinase inhibitor, Y27632 (10****µM: control hyperpolarization to SLIGRL, 26.2±3.14 mV, plus Y27632 26.5±4.2 mV, n = 8), despite a trend toward block, TRAM-34 did not significantly inhibit EDH (16.2±1.9 mV, n = 8). However, the subsequent addition of apamin significantly inhibited EDH (to 6.9±1.0 mV, n = 6, P<0.05). The residual hyperpolarization was again inhibited further by iberiotoxin, as in the absence of Y27632 (3.4±0.2 mV, n = 5; Figure 2C). These results indicate that stimulation of TP receptors with U46619 inhibits K_Ca_2.3 function and that subsequent inhibition of Rho kinase restores or protects this component of hyperpolarization.

### Inhibiting Rho Kinase Restores the Input of K_Ca_2.3 to EDH in the Presence of a NO Synthase Inhibitor

**Figure 3:**
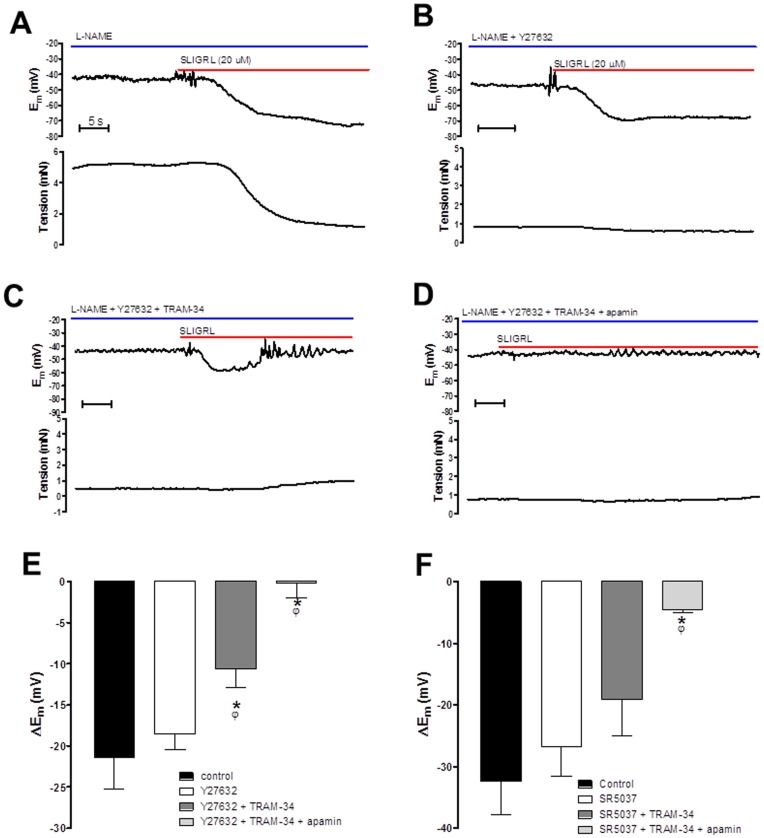
Original traces showing the isolated EDH response evoked by SLIGRL (20 µM) in rat MCAs treated with the NOS inhibitor L-NAME (100 µM). Upper panels show E_m_, lower panels tension. (A) Control EDH response. (B) EDH response in the presence of the Rho kinase inhibitor Y27632 (10 µM). (C) EDH response in the presence of Y27632 and the K_Ca_3.1 blocker, TRAM-34 (1 µM). (D) EDH response in the presence of Y27632, TRAM-34 and the K_Ca_2.3 blocker apamin (100 nM). (E) Histogram showing SLIGRL-induced EDH in the presence of Y27632 (10 µM), Y27632 and TRAM-34 and the combination of Y27632, TRAM-34 and apamin. (F) Histogram showing SLIGRL-induced EDH mediated hyperpolarization in the presence of SR5037 (1 µM), SR5037 and TRAM-34 and the combination of SR5037, TRAM-34 and apamin. Both Y27632 and SR5037 fully relaxed L-NAME induced tone, hyperpolarization was unaffected. Normally in the presence of L-NAME blockade of K_Ca_3.1 is sufficient to block the EDH response, however following inhibition of Rho kinase subsequent inhibition of K_Ca_2.3 is required to fully block the EDH response. *P<0.05 indicates a significant difference from control, one-way ANOVA with Tukey’s post-test, n = 4–6. ^φ^P<0.05 indicates a significant difference from Rho kinase inhibitor alone (Y27632 or SR5037) as determined by one-way ANOVA with Tukey’s post-test, n = 4–6.

Previous studies demonstrate that in the presence of the NOS inhibitor L-NAME (100 µM) the MCA constricts and EDH is blocked by TRAM-34 alone indicating a critical role for K_Ca_3.1 [1]. In this study 100 µM L-NAME contracted cerebral arteries and a higher concentration of L-NAME (300 µM) had no additional constrictor effect (data not shown). In the presence of L-NAME the EDH responses were completely inhibited by TRAM-34 alone (Figure S1) confirming that under these conditions only K_Ca_3.1 contributes to EDH. The contraction was reversed by SLIGRL (to stimulate EDH and associated relaxation of 21.4±3.9 mV and 67.9±6.1%, respectively n = 6; Figure 3A, E). Addition of the Rho kinase inhibitor Y27632 (10 µM) also reversed the L-NAME induced constriction (relaxation of 86.0±2.4%, n = 6) and the subsequent relaxation to SLIGRL became too small to assess accurately (Figure 3B). In contrast, EDH was unaffected by Y27632 (18.5±1.9 mV to 20 µM SLIGRL, n = 5), and TRAM-34 now only partially reduced this hyperpolarization (10.6±2.2 mV, n = 4, P<0.05; Figure 3C,E). The residual hyperpolarization was abolished by the additional presence of apamin (0.2±1.8 mV, n = 4, P<0.05; Figure 3D, E). A structurally distinct and extremely selective Rho kinase inhibitor SR5037 [20] had a similar effects to Y27632. This novel agent reversed vasoconstriction to L-NAME (relaxation of 85.2±3.7% n = 4) and the remaining tension was too small to assess accurately. SR5037 also restored the K_Ca_2.3 component to EDH, as TRAM-34 failed to completely block hyperpolarization and the combination of TRAM-34 and apamin were both required to block hyperpolarization (Figure 3F). Thus, in the presence of L-NAME block of K_Ca_3.1 with TRAM-34 alone inhibits the EDH response. Inhibition of Rho kinase with selective and structurally distinct inhibitors reveals a K_Ca_2.3 input to EDH responses indicating that Rho kinase normally supresses the function of these channels in the presence of L-NAME.

### Simvastatin Prevented Loss of K_Ca_2.3 Input to EDH Following TP Receptor Stimulation

**Figure 4:**
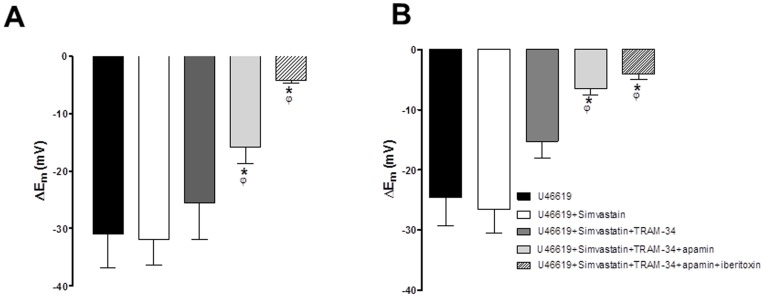
Histograms showing EDH evoked by SLIGRL (20 µM) in rat MCAs that are able to synthesise NO but treated with the TP receptor agonist, U46619 (50–100 nM). Also shown is the effect of the HMG-CoA reductase inhibitor simvastatin at either 100 nM (A) or 1 µM (B) on EDH and the effect of blocking K_Ca_3.1 alone (TRAM-34, 1 µM), in the subsequent presence of blockade of both K_Ca_2.3 (apamin 100 nM) and K_Ca_3.1 as well as the combined of blockade of K_Ca_1.1 (iberiotoxin 100 nM), K­_Ca_2.3 and K_Ca_3.1. *P<0.05 indicates a significant difference from control using one way ANOVA with Tukey’s post-test, n = 4–7. ^φ^P<0.05 indicates a significant difference from simvastatin (100 nM or 1 µM) as determined by one-way ANOVA with Tukey’s post-test, n = 4–7.

In MCAs able to synthesise NO inhibition of Rho kinase with a selective inhibitor protected K_Ca_2.3 function against TP receptor stimulation produced by U46619. In a separate group of experiments in vessels able to synthesise NO and stimulated with U46619 (50–100 nM) we assessed if statins shared this effect of Rho kinase inhibitors. SLIGRL (20 µM) evoked EDH of 30.9±5.9 mV (n = 7). This response was not modified by 100 nM simvastatin (31.8±4.4 mV, n = 7). Subsequently, EDH was not reduced with TRAM-34 (25.4±6.4 mV, n = 4), but was progressively and significantly reduced by adding apamin and then iberiotoxin EDH (to 15.8±2.9 mV, n = 4, P<0.05 then 4.1±0.5 mV, n = 3, P<0.05; Figure 4A). At a higher concentration simvastatin (1 µM) again did not modify EDH evoked in the presence of U46619 (at 24.6±4.6 mV and 26.6±4.0 mV, pre and post simvastatin respectively, n = 8), and a similar inhibitory profile was observed with K_Ca_ channel blockers, EDH of 15.2±2.9 mV, n = 8 with TRAM-34, 6.5±1.0 mV, n = 5, P<0.05 with apamin and 4.0±0.9 mV, n = 5, P<0.05 with iberiotoxin (Figure 4B). Simvastatin (100 nM or 1 µM) did not significantly alter U46619 induced tone; however a higher concentration of simvastatin (10 µM) fully relaxed tone (data not shown). Normally there is no K_Ca_2.3 component to hyperpolarization in the presence of U46619 but in the presence of simvastatin, at either concentration, blockade of this channel was required to significantly inhibit hyperpolarization. Thus a statin, like the Rho kinase inhibitor, protects the K_Ca_2.3 component to EDH from TP receptor stimulation.

### Simvastatin and Lovastatin Restore the Input of K_Ca_2.3 to EDH in the Presence of a NO Synthase Inhibitor while Geranylgeranyl-pyrophosphate Circumvents this Protection

**Figure 5:**
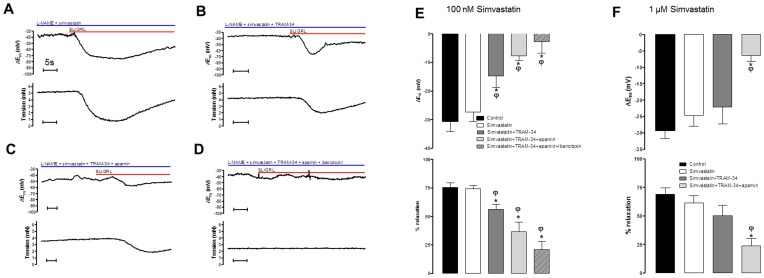
Effect of simvastatin on isolated EDH responses obtained in the presence of a NO synthase inhibitor. (A–D) Original traces showing the effect of 100 nM simvastatin (A) on isolated SLIGRL-induced EDH-mediated responses (hyperpolarization, upper panels; relaxation, lower panels) obtained in rat MCAs treated with the NOS inhibitor L-NAME (100 µM). Also shown is the effect of block of K_Ca_3.1 (TRAM-34; B); combined block of K_Ca_2.3 and 3.1 with apamin and TRAM-34 (C) and the further blockade of K_Ca_1.1, 2.3 and 3.1 with iberiotoxin, apamin and TRAM-34 (D). Also shown (E–G) are histograms of the mean data for SLIGRL-induced EDH mediated responses (hyperpolarization, upper panels; relaxation, lower panels) in the presence of 100 nM simvastatin (E), 1 µM simvastatin (F). Normally in the presence of L-NAME inhibition of K_Ca_3.1 alone is sufficient to block the EDH response. However, statins revealed a K_Ca_2.3 component to the EDH response. *P<0.05 indicates a difference from control, ^φ^P<0.05 indicates a significant difference from simvastatin (100 nM or 1 µM) as determined by one-way ANOVA with Tukey’s post-test, n = 5–8.

In rat MCAs treated with L-NAME we assessed if statins shared the effect of Rho kinase inhibitors to restore the K_Ca_2.3 component of EDH. EDH and relaxation to SLIGRL was 30.7±3.5 mV and 75.5±4.1%, respectively (n = 8 Figure 5E). In the presence of 100 nM simvastatin this was unchanged (28.2±3.8 mV, n = 7, and 74.2±3.0%, n = 8; Figure 5A, E). TRAM-34 then significantly attenuated hyperpolarization and relaxation (to 14.8±4.0 mV and 56.4±4.2%, respectively, n = 8, P<0.05; Figure 5B, E). The subsequent addition of apamin further attenuated these responses (to 7.7±1.6 mV and 36.5±8.5%, n = 6, P<0.05; Figure 5C,E) as did iberiotoxin (to 2.7±3.9 mV and 21.1±7.0%, n = 4, P<0.05; Figure 5D, E). Both 1 µM simvastatin (figure 5F) or 100 nM Lovastatin (Figure S2) had a similar influence i.e. a combination of TRAM-34 and apamin was required to significantly to block the EDH response (Figure 5F and Figure S2). Normally in the presence of L-NAME, TRAM-34 alone can block the EDH response (Figure S1). In the presence of statins the additional requirement of apamin to fully inhibit EDH indicate these agents share the ability of Rho kinase inhibitors to protect K_Ca_2.3 function.

**Figure 6:**
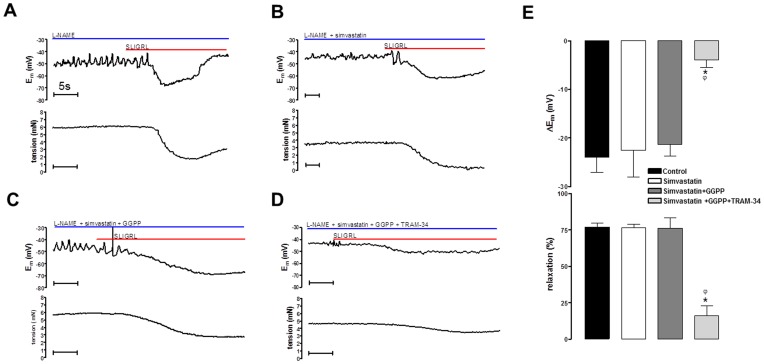
Effect of restoring the isoprenoid signalling pathway on EDH responses obtained in the presence of simvastatin and a NO synthase inhibitor. (A–D) original traces showing isolated SLIGRL-induced, EDH-mediated hyperpolarizations (upper panels) and relaxations (lower panels) obtained from rat MCAs treated with the NOS inhibitor L-NAME (100 µM; A). Also shown is the additional effect of simvastatin (100 nM), addition of GGPP (1 µM; C) and the combination of simvastatin and GGPP with the K_Ca_3.1 inhibitor TRAM-34 (1 µM; D). (E) Histogram showing the mean data for isolated EDH-mediated responses (hyperpolarization, upper panel; relaxation, lower panel). While GGPP did not alter the total EDH mediated response it reversed the ability of simvastatin to protect K_Ca_2.3 function as inhibition of K_Ca_3.1 with TRAM-34 alone was sufficient to significantly inhibit the EDH response. *P<0.05 indicates a significant difference from control using one-way ANOVA with Tukey’s post-test, n = 4.^ φ^P<0.05 indicates a significant difference from simvastatin, as determined by one-way ANOVA with Tukey’s post-test, n = 4.

Further experiments were conducted to assess the potential mechanisms by which statins protect K_Ca_2.3 function in arteries treated with L-NAME. EDH and relaxation to SLIGRL (23.9±3.2 mV and 76.9±2.9%, n = 4) was not altered by simvastatin (100 nM; 22.5±5.5 mV and 76.6±2.3%, n = 4) or by the combination of simvastatin and isoprenoid GGPP (1 µM; 21.3±2.4 mV and 76.2±7.1%, n = 4). However, with GGPP present EDH responses were virtually abolished by the addition of TRAM-34 alone (3.9±1.6 mV and 16.3±6.5%, n = 4, P<0.05; Figure 6). As GGPP only restores the isoprenylation pathway and not cholesterol synthesis [28] the ability of statins to protect K_Ca_2.3 function is likely to be a pleiotropic effect independent of effects on cholesterol synthesis.

## Discussion

These data implicate a novel role for Rho kinase in mediating TP receptor-dependent regulation of K_Ca_2.3 channel function in EDH and associated smooth muscle cell relaxation in the rat MCA. They provide a mechanistic explanation for our previous observation that NO can protect K_Ca_2.3 function in this artery by suppressing TP receptor signalling [6] and that stimulation of TP receptors can inhibit K_Ca_2.3 function [6], [7], [29]. Our data also show that statins [21] restore K_Ca_2.3 input to EDH and that this action is independent of effects on synthesis of cholesterol, possibly reflecting inhibition of Rho-mediated signalling.

To record and quantify the EDH response it is necessary to block NOS. In most arteries inhibition of EDH requires combined block endothelial cell K_Ca_2.3 and 3.1 channels [3] Unusually, the rat MCA displays an EDH response that is solely due to K_Ca_3.1 activation, as inhibition of K_Ca_3.1 abolished EDH regardless of the agonist used to stimulate the EDH response [1], [2]. However, MCAs express both K_Ca_2.3 and K_Ca_3.1 channel protein [4], [5]. An explanation for this apparent paradox is that K_Ca_2.3 can contribute to EDH, but only when the artery is able to synthesize NO, i.e. in the absence of a NOS inhibitor [5]. K_Ca_2.3 also contributes to EDH in the presence of a NOS inhibitor, provided that TP receptors are antagonized [6]. In each case, inhibition of K_Ca_2.3 alone had no discernible effect, but inhibition of EDH was now only significant following inhibition of *both* K_Ca_2.3 and K_Ca_3.1. The mechanism by which TP receptors selectively suppressed K_Ca_2.3 function was not clear.

TP receptors couple to several second messenger systems, particularly PLC [30], and small GTPases such as Rho [31], [32]. In vascular smooth muscle cells, both these pathways contribute to constriction, but Rho-mediated pathways predominate [13], block of U46619-mediated constriction confirms this in the MCA. We demonstrate that TP receptors are also located on endothelial cells of the MCA (Figure 1). As K_Ca_2.3 are only expressed in the endothelium of this artery [5], TP receptors could potentially selectively regulate the function of this channel by Rho kinase-dependent mechanisms.

**Figure 7:**
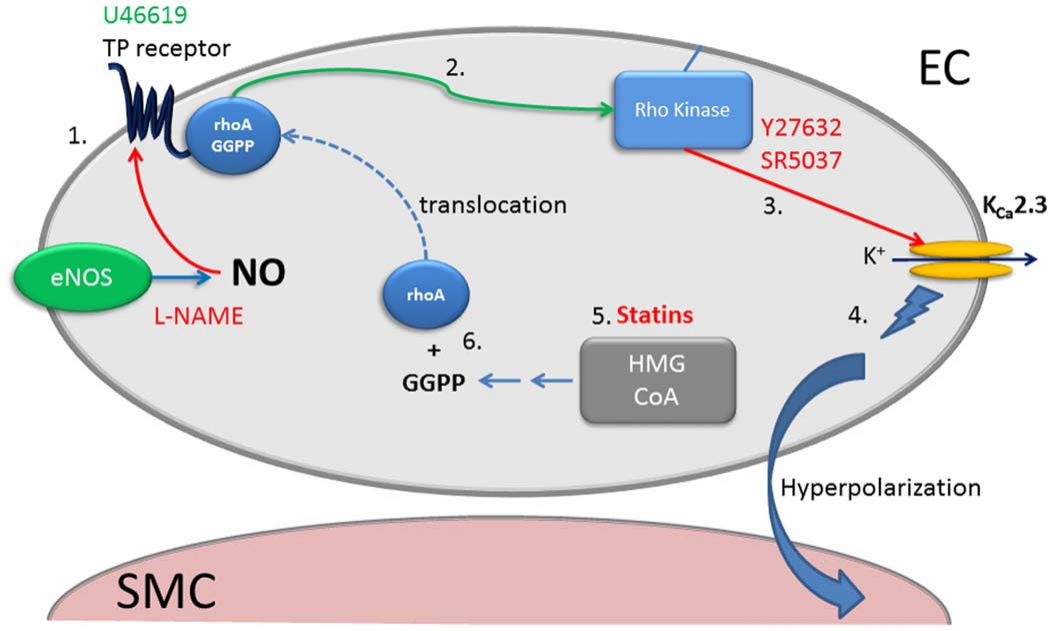
Schematic diagram showing potential regulatory mechanisms of K_Ca_2.3 channels by TP receptor/rho mediated signalling. Increased stimulation of the TP receptor with the agonist U46619 or following inhibition of NOS (eNOS) with L-NAME (NO can supress the action of TP receptors or synthesis of metabolites that activate TP) (**1**) results in activation of rhoA and stimulation of Rho kinase (**2**). Rho kinase (or associated signalling) inhibits K_Ca_2.3 function (**3**) and inhibitors of Rho kinase (Y27632 or SR5037) restore or protect the K_Ca_2.3 component of EDH (**4**). Statins by inhibiting HMG-CoA prevent formation of the isoprenoid GGPP (**5**). This reduces Rho mediated signalling by preventing GGPP dependent translocation of rhoA to the plasma membrane (**6**). Therefore statins protect K_Ca_2.3 function by inhibiting Rho-mediated signalling via the TP receptor (**1–3.**). Red arrows/text indicates an inhibitory mechanism. Green text/arrows represent a stimulatory mechanism. Blue arrows indicate synthetic pathways; dashed blue line indicates translocation to the plasma membrane.

We assessed the role of Rho kinase in regulation of K_Ca_2.3 channels in MCAs able to synthesise NO. Under these conditions stimulation of TP receptors causes a loss of function of K_Ca_2.3 channel [6]. We now show that inhibiting Rho kinase with Y27632 can “protect” or restore the K_Ca_2.3 input to EDH that is blocked in the presence of the TP receptor agonist, U46619 (Figure 2C). It is important to note that the residual K_Ca_1.1 component of EDH seen in these conditions is NO mediated [10]. In the absence of NO synthesis (i.e. in the presence of L-NAME), EDH was blocked with TRAM-34 (Figure S2), indicating that K_Ca_3.1 channels alone underpinned hyperpolarization, as previously reported [1]. In the presence of either Y27632 or a structurally distinct and highly selective Rho kinase inhibitor, SR5037 [20], TRAM-34 now failed to fully block EDH (Figure 3); EDH was only fully blocked if K_Ca_2.3 channels were subsequently inhibited with apamin (Figure 3D–F). It was impossible to measure effect on EDH mediated relaxation as Rho kinase inhibition fully relaxed tone (Figure 3B). Thus, inhibition of Rho kinase restores the K_Ca_2.3 component of EDH, indicating that TP signalling through Rho kinase negatively and selectively modulates K_Ca_2.3 (Figure 7). Inhibition of Rho kinase also reduced the constriction produced by blockade of NOS implying that Rho kinase is involved in this response, which is perhaps unsurprising since TP receptor stimulation is implicated in the constriction produced by NOS inhibitors in the MCA [33], [34].

As Rho kinase appears to inhibit endothelial cell K_Ca_2.3 function, we postulated statins might act to ‘protect’ the input of these channels in a similar manner to the Rho kinase inhibitors. As a consequence of inhibiting HMG-CoA reductase, statins also inhibit Rho kinase [35]. This “pleiotropic” effect is explained by an ability to prevent isoprenylation and subsequent translocation to the membrane of GTPases, such as Rho, an effect reversed by addition of exogenous isoprenoids [15], [23]. This appeared to be the case, in arteries able to synthesise NO and exposed to the TP receptor agonist U46619 (to inhibit K_Ca_2.3) the statin, simvastatin (100 nM and 1 µM) restored the input of K_Ca_2.3 to EDH (Figure 4). Simvastatin also restored K_Ca_2.3 input to EDH-mediated responses once NOS was blocked (Figure 5A–F); a response that normally reflects only the activity of K_Ca_3.1 (Figure S1) [1]. A structurally distinct statin, lovastatin had similar effects (Figure S2). At 100 nM simvastatin seemed to reveal a K_Ca_1.1 component to EDH that was absent at a higher concentrations or with lovastatin. NO synthesis was inhibited in these experiments so this probably reflects an ability of this lower concentration of simvastatin to directly stimulate this channel [36].

The restorative action of the statins on K_Ca_2.3 function is unlikely to reflect a reduction in cholesterol synthesis as this mainly occurs in the liver [28]. However, arteries do express HMG-CoA reductase [37], inhibition of which reduces production of isoprenoids including geranylgeranylpyrophosphate (GGPP) [28]. GGPP acts as a post-translational lipid attachment essential for the function of many proteins including Rho, rac and cd42 [28], [38]. Exogenous GGPP circumvented the ability of statins to restore K_Ca_2.3 function (Figure 6). Therefore, statins protect K_Ca_2.3 channel function by inhibiting isoprenylation. As reductions in isoprenylation reduce Rho signalling we speculate this is the mechanism by which statins protect K_Ca_2.3 function. Indeed, inhibition of Rho is the mechanism by which statins augment NOS function in endothelial cells [15], [23]. Although the concentrations of statins used in this study did not produce powerful vasodilator responses seen with the inhibitors of Rho kinase, higher concentrations of statins (>1 µM) do cause relaxation (data not shown). Perhaps this indicates that the lower, clinically relevant concentrations [21] used in this study are not sufficient to fully inhibit Rho signalling in smooth muscle cells.

It is important to note we used the closed ring (lactone) form of simvastatin (and lovastatin) in this study. These require hydrolysis to the open ring Na^+^ salt (acid) form to inhibit HMG-CoA. [39] a process that occurs rapidly in rodent plasma [40]. While it is unknown if it this can also occur in arterial tissues CYP450 enzymes can dehydrogenate lovastatin (and simvastatin) [41] to the open acid form and the CYP3A4 subtype involved has been shown to be expressed in monkey endothelial cells [42]. Regardless, in this study GGPP reversed the effects of the lactone version of simvastatin indicating that the effects were mediated via inhibition of HMG Co-A. This is supported by a previous report in porcine vascular smooth muscle cells where the lactone form of simvastatin mediated effects dependent on inhibition of HMG Co-A [36].

Although our data strongly suggest a regulatory role for rho kinase on endothelial cell K_Ca_2.3 channel input to EDH, it remains to be determined how Rho/Rho kinase inhibits K_Ca_2.3. It is possible regulation involves direct phosphorylation of the channel by Rho kinase, or an associated kinase, similar to the regulation of K_Ca_3.1 channels by kinases in immune cells [43]. Alternatively, Rho kinase might indirectly modulate K_Ca_2.3 by remodelling the cytoskeleton; delayed rectifier channels in cerebral artery smooth muscle cells are inhibited by actin polymerization that is Rho kinase dependent [44].

Whatever the precise mechanism, vasoconstrictors such as TP, endothelin-1 and angiotensin II signal via the Rho kinase pathway and receptors for these vasoconstrictors are present on endothelial cells [14], [45], [46]. As a number of models of hypertension and diabetes are associated with an increased production/function of these vasoconstrictors it is perhaps not surprising that EDH-mediated signalling is suppressed [47]. The ability of statins to protect endothelial cell K_Ca_ function appears to involve isoprenoids and Rho-mediated signalling; such protection may help to explain the beneficial effect of statin treatment after stroke [48]. As Rho signalling inhibits a vital component of EDH, K_Ca_2.3 channels located on the endothelium, our data support the rationale for treating endothelial dysfunction and related disorders by targeting Rho signalling.

## Supporting Information

Figure S1 **Histograms of the mean data for SLIGRL-induced EDH (hyperpolarization, upper panels; relaxation, lower panels) in the presence of the NOS inhibitor L-NAME (100 and 300 µM).** The EDH responses were completely abolished by the K_Ca_3.1 blocker TRAM-34 (1 µM). *P<0.05 indicates a significant difference from the 100 µM L-NAME control group; n = 3.
(TIF)

Figure S2 **Histograms of the mean data for SLIGRL-induced EDH mediated (hyperpolarization, upper panels; relaxation, lower panels) in the presence of the NOS inhibitor L-NAME (100 µM) and the subsequent effect of the statin lovastatin (100 nM).** Normally in the presence of L-NAME inhibition of K_Ca_3.1 alone is sufficient to block the EDH response. However, lovastatin revealed a K_Ca_2.3 component to the EDH response. *P<0.05 indicates a difference from control, ^φ^P<0.05 indicates a significant difference from lovastatin as determined by one-way ANOVA with Tukey’s post-test, n = 5.
(TIF)
