# Supplementary figures and images for: Statins and Selective Inhibition of Rho Kinase Protect Small Conductance Calcium-Activated Potassium Channel Function (KCa2.3) in Cerebral Arteries
Source: PLoS One. 2012 Oct 8;7(10):e46735. doi: 10.1371/journal.pone.0046735 (PMC3466297; doi:10.1371/journal.pone.0046735)

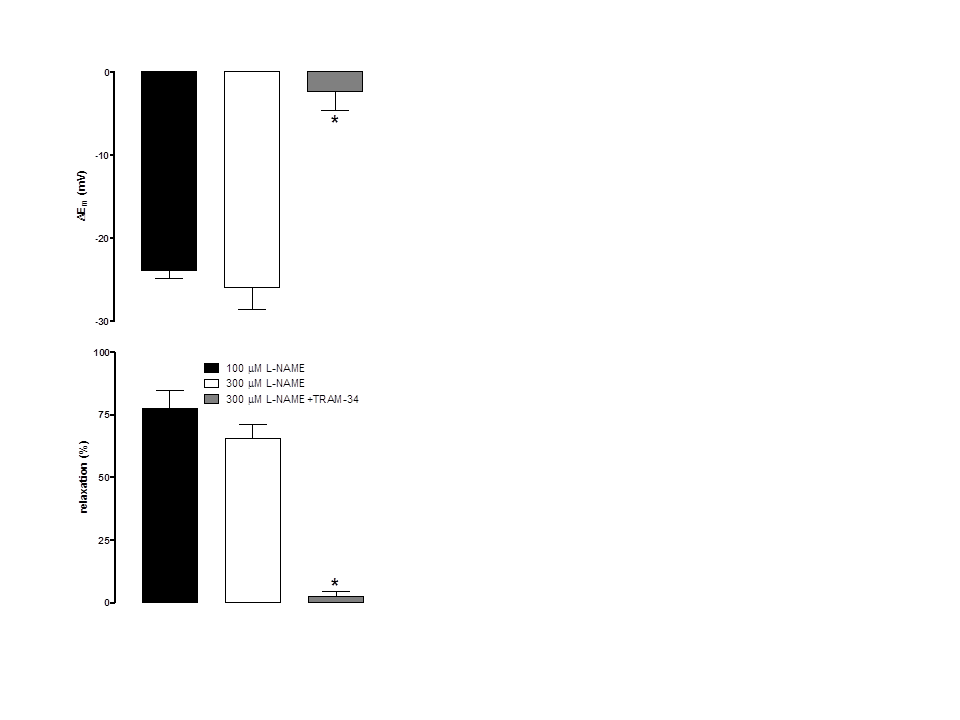

Supplement: Figure S1 — Histograms of the mean data for SLIGRL-induced EDH (hyperpolarization, upper panels; relaxation, lower panels) in the presence of the NOS inhibitor L-NAME (100 and 300 µM). The EDH responses were completely abolished by the KCa3.1 blocker TRAM-34 (1 µM). *P<0.05 indicates a significant difference from the 100 µM L-NAME control group; n = 3. (TIF) [file pone.0046735.s001.tif]

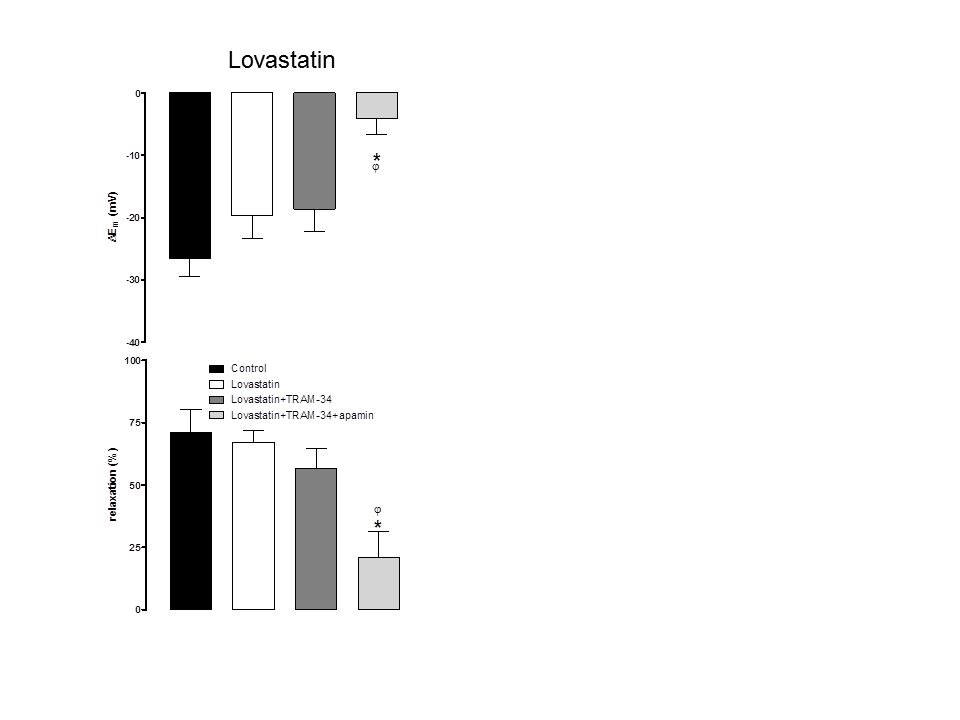

Supplement: Figure S2 — Histograms of the mean data for SLIGRL-induced EDH mediated (hyperpolarization, upper panels; relaxation, lower panels) in the presence of the NOS inhibitor L-NAME (100 µM) and the subsequent effect of the statin lovastatin (100 nM). Normally in the presence of L-NAME inhibition of KCa3.1 alone is sufficient to block the EDH response. However, lovastatin revealed a KCa2.3 component to the EDH response. *P<0.05 indicates a difference from control, φP<0.05 indicates a significant difference from lovastatin as determined by one-way ANOVA with Tukey’s post-test, n = 5. (TIF) [file pone.0046735.s002.tif]
